# A survey of global radiation damage to 15 different protein crystal types at room temperature: a new decay model

**DOI:** 10.1107/S0909049512049114

**Published:** 2012-12-06

**Authors:** Ricardo Miguel Ferraz Leal, Gleb Bourenkov, Silvia Russi, Alexander N. Popov

**Affiliations:** a ESRF, BP-220, 38043 Grenoble, France; b ILL, BP 156, 38042 Grenoble, France; c EMBL Hamburg Outstation, c/o DESY, Notkestrasse 85b, Hamburg 22607, Germany

**Keywords:** radiation damage, room temperature, X-ray diffraction, data collection

## Abstract

The radiation damage rates to crystals of 15 model macromolecular structures were studied using an automated radiation sensitivity characterization procedure. The diffracted intensity variation with dose is described by a two-parameter model. This model includes a strong resolution-independent decay specific to room-temperature measurements along with a linear increase in overall Debye–Waller factors. An equivalent representation of sensitivity *via* a single parameter, normalized half-dose, is introduced. This parameter varies by an order of magnitude between the different structures studied. The data show a correlation of crystal radiation sensitivity with crystal solvent content but no dose-rate dependency was detected in the range 0.05–300 kGy s^−1^. The results of the crystal characterization are suitable for either optimal planning of room-temperature data collection or *in situ* crystallization plate screening experiments.

## Abstract 2

A systematic study of the sensitivity to radiation damage of crystals held at room temperature for a large set of model macromolecular structures is presented.

## 1. Introduction   

The vast majority of macromolecular crystallographic data are collected at cryotemperatures. This is because the rate of overall decay in the diffraction intensity signal, also known as ‘global radiation damage’ (Holton, 2009 ▶), slows down by about two orders of magnitude at 100 K compared with at room temperature (RT) (Nave & Garman, 2005 ▶). Significant progress has been made in providing a quantitative phenomenological description of global radiation damage at cryotemperatures (reviewed by Holton, 2009 ▶; Garman, 2010 ▶). All macromolecular crystals that have been systematically examined to date show very similar radiation sensitivities. Therefore, the resolution-dependent intensity decay can be taken into account in the optimal planning of a diffraction experiment (Bourenkov & Popov, 2010 ▶). The remaining complications during cryogenic data collection arise mostly from the specific experimental conditions, in particular from a systematic mismatch of the beam size with crystal size at third-generation undulator beamlines (Krojer & von Delft, 2011 ▶). In contrast, the specific radiation-induced changes under cryo-conditions are often the source of severe difficulties in interpretation of structure and function (*e.g.* Dubnovitsky *et al.*, 2005 ▶; Borshchevskiy *et al.*, 2011 ▶).

Although infrequently used, RT data collection remains necessary for a number of studies. Cryogenic techniques introduce artifacts, either directly *via* temperature effects or indirectly because of the cryoprotectants and temperature-induced pH changes (Juers & Matthews, 2001 ▶; Halle, 2004 ▶; Dunlop *et al.*, 2005 ▶). In recent years, a renaissance of interest in RT experiments has occurred in the context of *in situ* diffraction methods where the entire crystallization plate is mounted on the beamline. A number of developments aiming at automation pursue the idea of *in situ* data collection in order to bypass the crystal handling step that is notoriously difficult to automate. Maire *et al.* (2011 ▶), Bingel-Erlenmeyer *et al.* (2011 ▶), Hargreaves (2012 ▶) and Axford *et al.* (2012 ▶) have all used the ‘in-plate’ screening and/or data collection approach. The concept of microfluidic-based crystallization also relies on RT ‘on-chip’ data collection (Hansen *et al.*, 2006 ▶; Dhouib *et al.*, 2009 ▶; Li & Ismagilov, 2010 ▶; Kisselman *et al.*, 2011 ▶). Recently, Axford *et al.* (2012 ▶) reported a series of case studies strongly supporting the *in situ* RT approach. Examples include the successful structure solution of bovine enterovirus-2, where successful cryoprotection of the crystals was not at all possible.

For the optimal design of the experiments described above, whether they involve screening or data collection, a prior knowledge of sample dose tolerance at RT is absolutely essential. Recent systematic studies (Southworth-Davies *et al.*, 2007 ▶; Barker *et al.*, 2009 ▶; Warkentin & Thorne, 2010 ▶; Warkentin *et al.*, 2011 ▶, 2012 ▶; Kmetko *et al.*, 2011 ▶; Rajendran *et al.*, 2011 ▶; Owen *et al.*, 2012 ▶) provided the global radiation damage data for a small number of structures under conditions of varying temperatures, chemical additives, dose rates and detector read-out frequencies. However, despite a long history of RT data collection from macromolecular crystals, practically no earlier data exist in a form suitable for systematic analysis and for making predictions on likely crystal dose tolerances.

In this work we investigate the global radiation damage rates of crystals of 15 different model structures, at RT and under native crystallization conditions. The principle aim of the experiments was to obtain a parametric description of diffraction intensity decay behavior suitable for data collection strategy optimization taking radiation damage into account using *BEST* (Bourenkov & Popov, 2010 ▶). The approach employed involves the collection of multiple partial data sets as a function of dose, and the use of a data analysis method developed specifically to verify the radiation damage model, to extract the model parameters and to minimize systematic errors. The reproducibility of the results was tested systematically. The experiments made use of the procedure for automatic characterization of radiation sensitivities at cryotemperatures developed earlier (Leal *et al.*, 2011 ▶). The further development and the modifications of the method that were required to account for the features of global radiation damage specific to RT are described.

## 2. Sample preparation and data collection   

A summary of the crystallographic parameters of the 15 crystal systems studied in this work is presented in Table 1 ▶. Different crystal forms of lysozyme, trypsin and insulin, as well as thaumatin, thermolysin and FAE, are standard test crystal systems. Crystal samples of bR, 6HLNO, LACV, TIM and TvNiR were kindly donated by the authors of the respective publications indicated in Table 1 ▶.

For data collection at RT, the open-flow humidity control apparatus HC1 was employed. The samples were prepared for measurements in the HC1 as described by Sanchez-Weatherby *et al.* (2009 ▶). The crystals were mounted on Mitegen micromeshes and excess liquid was removed with filter paper in order to prevent the crystals from moving during the data collection.

The measurements were carried out on ESRF beamline ID23-1 (Nurizzo *et al.*, 2006 ▶) using an ADSC Q315 detector. The X-ray beam energy was kept constant at 12.75 keV for all measurements. The nominal beam size at the sample position was 35 µm vertically and 45 µm horizontally (full width at half-maxima). In all cases crystals were selected such that all of the beam cross section was intercepted by the crystal.

The absorbed doses and rates were estimated with the program *RADDOSE* (Murray *et al.*, 2004 ▶; Paithankar *et al.*, 2009 ▶) using the photon flux values estimated by measurements with standard calibrated intensity monitors and the chemical composition of the protein, the ligands and the crystallization solution according to the reference in Table 1 ▶.

The standard procedure for radiation sensitivity measurements (Leal *et al.*, 2011 ▶), as implemented in *MxCuBE/EDNA* (Gabadinho *et al.*, 2010 ▶; Incardona *et al.*, 2009 ▶), was used. In this procedure the collection of 11 successive wedges of data interleaved by X-ray exposures to ‘burn’ the crystal are performed in a narrow rotation range (usually 3°), thus excluding variation in the exposed crystal volume. The procedure involves optimization of the intensity decay measurements, and necessarily requires preliminary knowledge or an assumption of the radiation sensitivity of the sample under consideration. Assuming that both the absorbed dose rate and the crystal sensitivity are known approximately, the burning and data collection protocol is generated automatically by the program *BEST* (Bourenkov & Popov, 2010 ▶) on the basis of the data obtained from the initial sample characterization step. The protocol defines a complete set of the parameters required: exposure time, attenuator transmission, total rotation range, rotation range per frame, the resolution limit (*d*_min_) for data collections, and the exposure time for irradiation. A detailed description of the methods used in the protocol generation is given by Leal *et al.* (2011 ▶). Using the model assumptions, the diffraction resolution limit and the dose for the burning cycles are selected in such a way that significant changes in the scattered intensities are induced, and the intensity measurements remain statistically significant up to the last cycle of data collection. At RT, the exposures between the burning cycles induced substantial radiation damage and often no burning cycles were required. In the method described by Leal *et al.* (2011 ▶) for cryotemperatures, the maximum dose per exposure cycle was 0.1 MGy. For RT data collection the maximum dose per exposure cycle was chosen such that the expected increase in the *B*-factor did not exceed 1 Å^2^. This consideration, combined with the standard *BEST* calculation as described by Popov & Bourenkov (2003 ▶) and Bourenkov & Popov (2010 ▶), gives rise to a consistent choice of the resolution limit, exposure time and rotation width per frame.

The data collection protocols were initially generated under the assumption of 70-fold higher radiation sensitivity at RT as compared with cryogenic conditions; the factor was chosen according to the previous studies at RT (Nave & Garman, 2005 ▶; Kmetko *et al.*, 2011 ▶). This corresponds to a total exposure time of approximately 1–2 s with the unattenuated X-ray beam, depending on the crystal absorbance and incident flux (varying with the ESRF filling mode). The shortest exposure time permitted on the ID23-1 diffractometer is 0.1 s per frame; thus for most of the measurements attenuation of the beam intensity was necessary.

Preliminary on-line data analysis indicated a strong variation in the radiation sensitivity between the samples studied. For sensitive crystals, the data processing typically failed on the last wedges whereas, for less sensitive samples, significant reduction of the intensity (about 50%) was not reached. For all systems studied (excluding TIM), after adjusting the sensitivity parameters according to the initial estimates of the decay rates, the measurements were repeated several times, either on the same crystal, after translating unexposed parts of the crystal into the beam, or by using different crystals. Finally all the data, including those obtained during the initial cycles, were included in the analysis.

## 3. Data analysis and results   

### 3.1. Scaling   

The data analysis performed in these experiments aimed for a parametric statistical description of the scattering power of the crystal as a function of absorbed dose. The scaling procedure differs conceptually from the standard scaling that minimizes intensity differences between (predominantly strong) equivalent reflections, as is commonly carried out during data reduction (Kabsch, 2010 ▶; Evans, 2011 ▶).

Here the scattering power is described by an expectation value of the reflection intensity 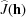 at a reciprocal lattice vector 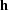. It can be expressed *via* a product of an empirical curve defining the radial shape 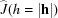 of the function, the scaling factor (*scale*) and the overall Debye–Waller factor (Popov & Bourenkov, 2003 ▶),
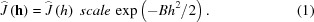
The function 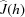 is defined by the interatomic distance distribution in macromolecules. The representation described above is widely used in current crystallographic methodology (*e.g.* Morris *et al.*, 2004 ▶; Adams *et al.*, 2010 ▶). Radiation damage does not lead to measurable changes in 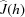. An empirical 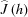 curve was tabulated as described (Popov & Bourenkov, 2003 ▶) and is on an arbitrarily chosen fixed scale. In the work presented here, use is made of the isotropic *B* factor approximation. Thus, equation (1) applies specifically to a narrow wedge of data, as in this experiment. With broader angular ranges, both the anisotropic *B* tensors and the variation in the *scale* parameter with crystal orientation (owing to the varying irradiated crystal volume) would have to be considered.

The parameters *scale* and *B* were estimated by maximizing the likelihood function [equation (18) of Popov & Bourenkov, 2003 ▶], as implemented in *BEST*. The function is derived under the assumption that the intensities obey the acentric Wilson distribution (Wilson, 1949 ▶). Lorentz-polarization-corrected un-scaled integrated intensities output by *XDS* (Kabsch, 2010 ▶) were used as the input to *BEST*. Thus, for each of the data wedges, the *scale* and *B* values were determined independently of the other wedges, and no scaling between wedges by *XDS* was involved. Applying the scaling procedure to the series of wedges measured on one crystal (or one crystal centering) gave the scaling parameters as a function of dose, *scale*(*D*) and *B*(*D*).

Compared with the standard scaling method, this approach does not rely on an assumption of identity in scaled intensities for sequential observations of the same 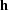. The latter assumption holds at low doses, when standard scaling may provide more accurate parameter estimates, but fails at progressively higher doses. The problems with the interpretability of the results of the standard *B*-factor scaling output in terms of radiation damage metrics have been discussed previously (Southworth-Davies *et al.*, 2007 ▶; Barker *et al.*, 2009 ▶; De la Mora *et al.*, 2011 ▶).

The dependence of the scaling results on the choice of the integration software (*XDS*, *HKL2000* or *MOSFLM*) has been analyzed previously in the course of testing the *BEST* software (data not shown). No systematic issues were identified.

### 3.2. Dose dependence of the *B*-factor   

**Figure 1:**
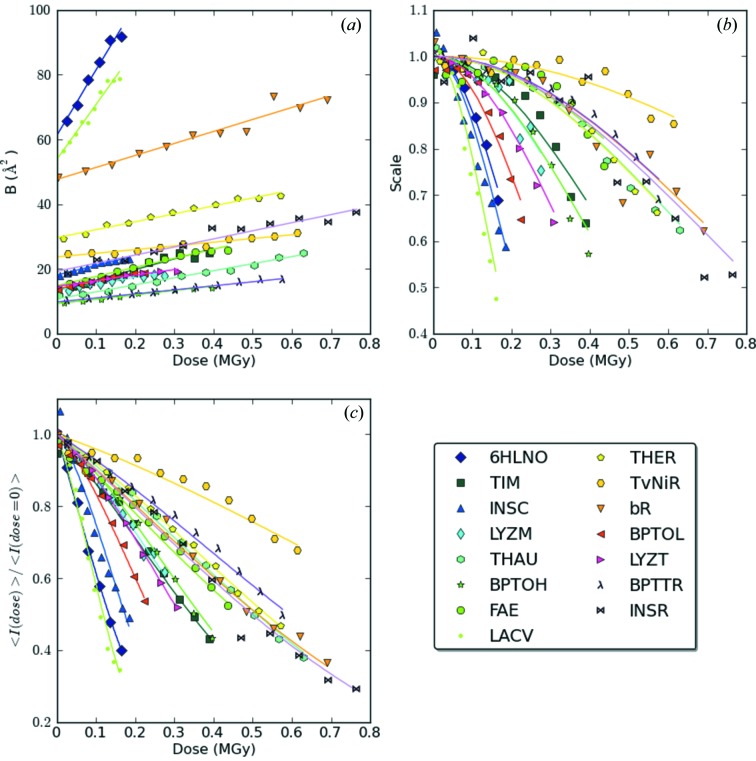
Variation in the scaling parameters and total scattering intensity with dose. For each of the systems studied, one measurement is shown. (*a*) *B*-factors: the solid line represents an approximation according to equation (2) using the best fit of *B*_0_ and β. (*b*) *scale* factors divided by the constant [equation (3)]: the solid line represents exp(−γ^2^ *D* ^2^) using the best fit value of γ. (*c*) Total scattered intensity, estimated by intensity summation (dots) and calculated according to equation (4) (solid lines), using the best fit *B*_0_, β and γ as in (*a*) and (*b*).

The dependence of the *B*-factor on the absorbed dose is presented in Fig. 1(*a*) ▶. For Figs. 1 (*a*), 1(*b*) and 1(*c*) ▶, for each of the systems studied, one measurement corresponding to the highest total absorbed dose was selected. The reproducibility of the experiments is shown in the supplementary figures^1^ where all the measurements are included.

As described in previous studies (Kmetko *et al.*, 2006 ▶; Bourenkov & Popov, 2010 ▶; Borek *et al.*, 2010 ▶; Warkentin & Thorne, 2010 ▶) carried out at both cryo or room temperatures, the observed distributions of the *B*-factor *versus* dose can be fitted by a linear function,
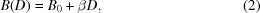
and the parameter β can be used as one of the radiation sensitivity characteristics. The average β values observed for each structure and their standard deviations are listed in Table 1 ▶ (see also the supplementary material). Whereas at cryogenic temperatures much lower values of β, varying within a narrow range of 0.5–1.2 Å^2^ MGy^−1^, were observed (Kmetko *et al.*, 2006 ▶; Leal *et al.*, 2011 ▶), at RT β values are higher by one to two orders of magnitude and show a large variation between structures.

### 3.3. Dose dependence of the *scale* factor   

The dependence of the *scale* factor on the absorbed dose is shown in Fig. 1(*b*) ▶. Under cryoconditions, only a small and approximately linear reduction in the *scale* factors has been observed, accounting for at most 5–15% of the overall drop in the intensity (Leal *et al.*, 2011 ▶). At RT, the decrease in the *scale* factor was pronouncedly non-linear, larger in magnitude and varied strongly between different structures. Empirically, the function
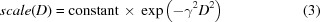
was found to describe the character of the distribution of the *scale* factor *versus* dose reasonably accurately, within the range of the *scale* factors sampled by our experiments. The constant and the parameter γ (Gy^−1^) have been fitted for all data sets (supplementary figures and table); the statistics for γ per structure are given in Table 1 ▶. Equivalent analysis of the cryotemperature data from Leal *et al.* (2011 ▶) showed the variation of γ to be between 0.03 and 0.05 MGy^−1^.

### 3.4. Dose dependence of the total scattered intensity   

Several recent radiation damage studies (*e.g.* Kmetko *et al.*, 2011 ▶) have used β as the only metric of radiation damage. With negligibly small γ values at cryotemperatures, such an approach is justified. However, neglecting the strong variation in overall scale factor at RT leads to a systematic underestimation of radiation sensitivity. As discussed, a two-parameter model (β and γ) is necessary and sufficient to construct a predictive model of resolution-dependent intensity variation. However, the use of a two-parametric model as a comparative metric relating the radiation sensitivity of different structures, or one and the same structure under various conditions, is extremely inconvenient.

The *D*_1/2_ parameter, the dose at which the total diffraction intensity reduces by a factor of two, was introduced as a radiation sensitivity metric by Garman and co-workers (Owen *et al.*, 2006 ▶) and used in a similar form in many subsequent studies (*e.g.* Barker *et al.*, 2009 ▶; Sanishvili *et al.*, 2011 ▶; Owen *et al.*, 2012 ▶).

The relationship between the dose-dependent scaling factors and the total scattered intensity may be derived in a straightforward way. Combining expressions (1), (2) and (3) and integrating over *h*, we obtain expression (4) for the total scattered intensity as a function of absorbed dose,
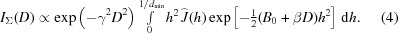
The total recorded intensities, estimated by the direct summation of all integrated intensities (corrected by the Lorenz-polarization factor, within the resolution limit *d*_min_), as a function of dose are shown in Fig. 1(*c*) ▶ (see supplementary figures for all data sets). The solid lines represent the *I*_Σ_(*D*)/*I*_Σ_(0) functions calculated using (4), the fitted values of γ, β and *B*_0_, and the respective values of *d*_min_ for each experiment. The calculated curves match the observations rather well. Corresponding half-dose values *D*_1/2_, satisfying the equation 2*I*_Σ_(*D*_1/2_) = *I*_Σ_(0), are listed in the supplementary table for each measurement.

### 3.5. Normalized half-dose   

**Figure 2:**
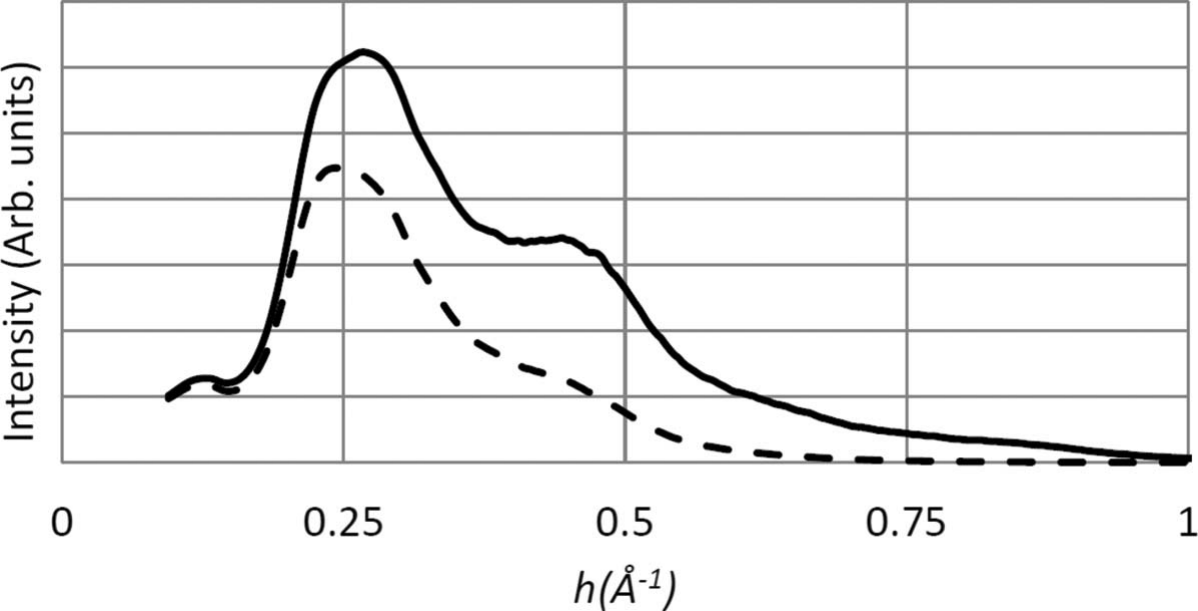
A generic empirical model of total diffraction intensity *versus* resolution, 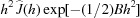, for *B* = 10 Å^2^ (solid line) and *B* = 20 Å^2^ (dashed line).

Two examples of the integrand function in equation (4) are given in Fig. 2 ▶ for (arbitrarily chosen) values of *B*_0_ + β*D* = 10 Å^2^ and 20 Å^2^. In all calculations presented here, the low-resolution integration limit was set at 1/12 Å, from the available 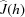 tabulation. This low-resolution truncation had no effect on the analysis.

The integration in (4) can be carried out either to the resolution limit of the data or until the integral converges. The first method corresponds to the regular procedure of determining *D*_1/2_ by direct summation of measured intensities, the second provides the true estimate of the total scattered intensity independent of the resolution of the experiment. It was found that the high-resolution integration cut-off had a significant systematic influence on the estimated *D*_1/2_ values. The *D*_1/2_ obtained by either the direct intensity summation or by the integration (4) within the same resolution limits are systematically higher (hence, the radiation sensitivity is underestimated) when compared with the estimates of *D*_1/2_ calculated by integrating (4) to convergence. For the THAU and FAE data, where the errors were largest, this amounts to a difference of about 30%.

Furthermore, from consideration of equation (4), Fig. 2 ▶ and the supplementary table, the systematic dependence of *D*_1/2_ on *B*_0_ is apparent. *D*_1/2_ is systematically larger for more weakly diffracting crystals having large *B*_0_, simply due to the fact that high-angle reflections, which fade out faster, are not present in those diffraction patterns even at the start.

In order to correct for this effect, we defined a new radiation sensitivity metric, the normalized half-dose, 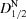. It is fully analogous to *D*_1/2_ but calculated using a standard value of *B*_0_ = 20 Å^2^ in equation (4) for all data sets, instead of the observed *B*_0_ value. This is equivalent to determining *D*_1/2_ by direct summation of intensities after their apodization to a standard overall *B*-factor of 20 Å^2^, but the effects of the experimental errors are avoided. Furthermore, 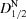 is determined by integrating (4) to convergence, thus the systematic errors related to the choice of data collection and/or processing resolution are excluded. The accuracy of 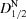 is mostly defined by the accuracy of β and γ; thereby an (anti-)correlated fraction of their error cancels in the product of the two exponentials contributing to (4). A reduction in the relative standard deviations of 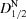 compared with either the β or γ parameter (Table 1 ▶) confirmed the considerations discussed above.

### 3.6. Radiation damage sensitivity correlation with solvent content   

**Figure 3:**
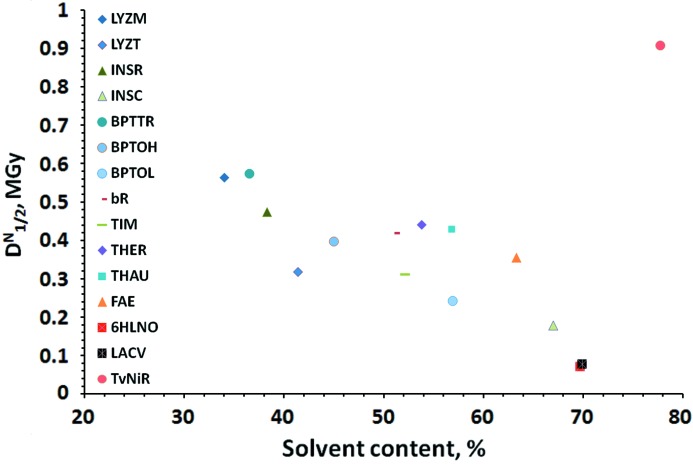
Normalized half-dose 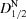 *versus* the crystal solvent content. For each of the systems studied, the markers represent the measured values averaged over all experiments performed.

The normalized half-dose parameter 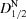 varied by more than an order of magnitude between different structures (Table 1 ▶). Attempts were made to correlate the observed sensitivities with many properties (chemical, physical and structural) of the proteins and the crystallization solutions involved, mostly unsuccessfully (data not shown). The only correlation observed in the data with a degree of confidence is that with crystal solvent content, Fig. 3 ▶. The general trend is higher sensitivity with increased solvent content. A change of a factor of six is observable between LZM with 34% solvent content to 6HLNO and LACV with 70% solvent. TvNiR crystals with 78% solvent were unusually radiation-hard and are thus an outlier: this is discussed below.

Considering separately the different crystal forms of lysozyme (LYZM, LYZT), insulin (INSR, INSC) and trypsin (BPTTR, BPTOH, BPTOL), higher solvent content strictly corresponds to lower 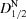 and therefore higher sensitivity. Three crystalline forms of trypsin grown under identical conditions in the same crystallization drop were measured; the solvent content and crystal packing is the only difference between them.

The residual variation unaccounted for by the different solvent contents is still significant. Interestingly, the LZT crystals, which by anecdotal evidence are usually considered radiation-hard, appear to be about 30% more sensitive than other structures with similar solvent content. In contrast, the only integral membrane protein involved in the analysis, bR, is a factor of two less radiation sensitive than trypsin.

### 3.7. TvNiR is an outlier   

In these experiments at RT, the TvNiR crystals exhibited abnormal radiation hardness. At 100 K, the radiation sensitivity of TvNiR is similar to all the other systems we examined (A. Popov, unpublished data), 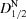 = 15–20 MGy. At RT, its normalized half-dose is higher by an order of magnitude when compared with that expected for a solvent content of 78%. It is also almost twice as resistant as the most radiation-hard low-solvent crystal forms of lysozyme and insulin. TvNiR is an octaheme cytochrome *c* nitrite reductase form of haloalkaliphilic bacterium (Polyakov *et al.*, 2009 ▶). It catalyzes the six-electron reduction of nitrite to ammonia or sulfite to sulfide, and the two-electron reduction of hydroxylamine ammonia; peroxidase activity of TvNIR has also been detected (Tikhonova *et al.*, 2006 ▶). It may be plausible to suppose that, under X-ray irradiation in the presence of an excess of one of its substrates, TvNiR would turn over thereby removing electrons and hydrogen, and thus acting as a very efficient scavenger. However, the TvNiR crystals used in this study were prepared in the absence of any known substrate. We speculate that TvNiR catalyzed the conversion of one or several water radiolysis products, *i.e.* peroxide, hydroxyl, hydroperoxyl or superoxide anion to water.

### 3.8. Dose-rate dependence of radiation damage   

**Figure 4:**
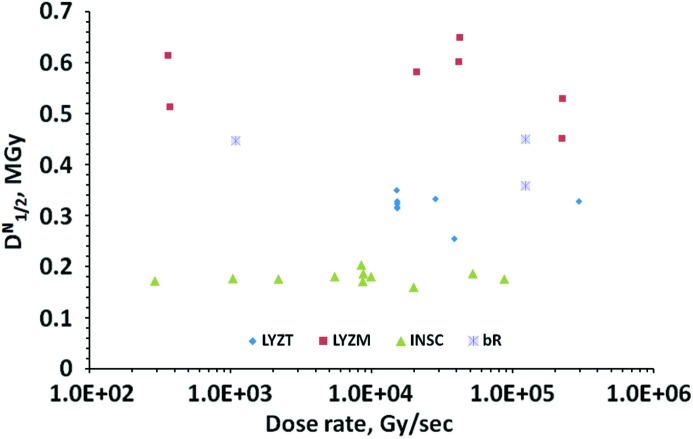
Normalized half-dose 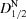 *versus* the dose rate for LYZT, LYZM, bR and INSC.

The measurement procedure unavoidably involved a large variation in the dose rates between each of the experiments (Table 1 ▶). This was due to the large range of radiation sensitivities observed among the test systems, the lower limit on the shortest exposure time imposed by the diffractometer, and the different synchrotron filling modes available during the experiments, which were performed over a period of several months. In order to exclude any misinterpretation arising from such variations, a series of measurements was carried out by deliberately attenuating the incident beam. The dose rate was varied over three orders of magnitude for LYZM, bR and INSC, and over an order of magnitude for LYZT. The results are presented in Fig. 4 ▶, which shows no systematic dependence of the normalized half-dose 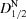 on the dose rate in any of the three experiments. For INSC, all the measurements were carried out on different parts of one large crystal and the distance between the irradiated spots was at least twice the beam size (full width at half-maximum). 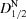 remained remarkably constant over the whole dose-rate range covered. The random noise is somewhat higher for LYZM and LYZT, where measurements were performed on different crystals.

## 4. Discussion   

### 4.1. Application of the workflow   

The reliable determination of the radiation sensitivity of macromolecular crystals is not a trivial experimental task. When assessing radiation damage *via* retrospective analysis of regular data sets collected in the process of structure solution, we are systematically confronted with a number of un­accountable critical details of the experiment (Krojer & von Delft, 2011 ▶). As demonstrated by Nowak *et al.* (2009 ▶), reproducibility issues are also evident in characterization experiments. We conclude that standard data collection protocols are not suitable for the measurement of radiation sensitivity. Therefore, an automated characterization procedure was developed and extensively tested under cryogenic conditions (Leal *et al.*, 2011 ▶). The procedure is currently implemented in an integrated workflow, transparently connecting experiment and data analysis at the ESRF beamlines (Brockhauser *et al.*, 2012 ▶). It has proven to be a convenient and reliable tool delivering reproducible data.

Here the workflow has been applied to a large set of model structures at RT. The use of the capillary-free mounting technique with the humidity control device HC1 (Sanchez-Weatherby *et al.*, 2009 ▶) simplified sample handling in these experiments. Furthermore, it excluded uncontrolled dehydration or crystal slippage.

### 4.2. An updated decay model   

The main purpose of the radiation sensitivity measurements was to estimate the parameters of a model describing resolution-dependent intensity variation with dose, in a form suitable for optimization of data collection in *BEST*. The model used in *BEST* was previously constructed on the basis of empirical data collected at cryotemperature, where a linear increase in overall *B*-factors is the totally dominant factor.

#### 4.2.1. The Debye–Waller factor model   

All the RT experiments presented here (77 decay curves in total) showed a linear increase in *B*-factor with dose, essentially up to the point where the loss of the signal-to-noise of high-resolution reflections made accurate determination of *B*-factors impossible. From multiple measurements, it is estimated that the relative error in determining the coefficient β is about 15% in this procedure. The average value of β = 20 Å^2^ MGy^−1^ for THAU is between those of Warkentin & Thorne (2010 ▶) who reported β = 35 Å^2^ MGy^−1^, and Rajendran *et al.* (2011 ▶) who reported β = 16 Å^2^ MGy^−1^. For INSC, β = 32 Å^2^ MGy^−1^ agrees broadly with the 44 Å^2^ MGy^−1^ reported (Rajendran *et al.*, 2011 ▶).

#### 4.2.2. The scale factor model   

The experiments described here indicate that due to the presence of a strong decay component which is independent of the scattering angle, the linear *B*-factor model alone significantly underestimates the intensity variation in all resolution shells at RT. The noise in the *scale*(*D*) dependence is significantly higher than that in *B*(*D*). This noise is ascribed to the variation in the three-dimensional spot profile parameters that are independently adjusted by *XDS* (Kabsch, 2010 ▶) for each processed wedge. Note that such adjustments would occur with any processing package; they have no negative effect on any of the standard crystallographic data set statistics. The accuracy of *scale*(*D*) is also a dominating factor defining the match of the *I*_Σ_(*D*) curves with the approximation in equation (4). Noticeable deviations of about 10% are observed specifically at low doses for several examples (*e.g.* INSC in the supplementary figures, panel *c*). We speculate that a sharp increase in the crystal mosaicity after the first exposures leads to a significant change in the integrated intensity values which are unaccounted for by the current analysis.

Nevertheless, at this noise level one can confidently see the concave character of the *scale*(*D*) dependency, with a linear decay rate 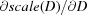 increasing with dose. The analytical function (3) chosen to model this dependence represents a simplest uni-parametric function satisfying the second-order kinetic equation
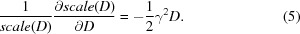
Equation (3) may only be considered an empirical parameterization useful to quantify the intensity decay within the range sampled by our experiments, typically *scale*(*D*) > 0.6. The nature of the variation in *scale*(*D*) and its concave shape is rather obscure. Within the framework of the standard crystallographic methodology, *scale* is proportional to the number of exposed unit cells and (3) could describe the formation of extended non-crystalline ‘void’ areas within the sample. The increase in the linear decay rate could then be connected to de-stabilization of the crystalline areas in the vicinity of the voids. In Hendrickson (1976 ▶) such a destabilization was proposed to be responsible for the time-dependent intensity decay component, but naturally not for the dose-dependent one. Thus, the model is at odds with the dose-rate independence of our experiments. So far it is clear only that the observed changes in the scale factors are connected to defects of the crystal lattice. At this point neither the exact type(s) of the defects involved and their relationship to the observed integrated intensities, nor the defect kinetics with the dose, can be assigned. Answering this question would require complex experiments unrelated to crystallographic data collection.

#### 4.2.3. Applications of the model   

Despite its unclear physical meaning, the γ parameter was determined reproducibly in multiple measurements with an r.m.s.d. error of approximately 20%. The decay model used in the program *BEST* in versions 3.4.4 and higher was adopted to make use of the two-parameter (β, γ) model. The accuracy of determining β and γ, as may be achieved in a ‘sacrificial crystal’ experiment, is clearly sufficient for the optimization of data collection parameters: the signal-to-noise ratio in the data as a function of the absorbed dose varies only slowly in the vicinity of its attainable maximum (Bourenkov & Popov, 2010 ▶). At the same time, neither 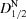 nor the *D*_1/2_ parameter alone would be sufficient to define an optimal exposure time or dose in a particular ‘sacrificial crystal’ experiment (due to the strong resolution dependence in both the decay rate and in the signal-to-noise ratio). One should note that the β, γ model and the optimization methods mentioned above are only applicable at a relatively high resolution. The lowest resolution data set presented here was at 4.0 Å (LACV). Presumably, a practical limit of applicability would be at around 4–5 Å. Real-life examples of optimized RT data collections using the two-parameter model will be published elsewhere. The sensitivity calibration at RT may require two iterations, since the sensitivity may deviate strongly from initial expectations.

### 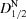 as a comparative metric   

4.3.

In order to represent the results of the sensitivity measurements of different types of crystals in a form suitable for comparison, the model based on two highly correlated parameters was numerically reduced to a single parameter 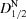, which is equivalent to the half-dose parameter previously used in many studies. Appropriate normalization removes severe systematic dependence on the experimental conditions and the initial diffraction power of the crystal, both of which are inherent to *D*_1/2_ determined by direct intensity summation. Note that to determine the half-dose our treatment uses the same number of fit parameters (two) as the direct summation method, but does not suffer from a systematically occurring inconsistency when *I*_Σ_(*D*)/*I*_Σ_(0) ≠ 1 at *D* = 0. We propose the use of the normalized 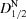 metric in radiation damage studies in general, or at least in those where decay dependence on the resolution is not an explicit subject of the experiment. The software for this analysis will be made available along with *BEST*.

For the LYST, INSC and THAU crystal systems, room-temperature radiation sensitivity data have been reported previously. Within the difference in the definitions and analysis, 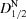 of LYST compare well with *D*_1/2_ reported by Barker *et al.* (2009 ▶) (0.3 *versus* 0.2 MGy), INSC (0.18 *versus* 0.2 MGy) and THAU (0.4 *versus* 0.35 MGy) by Rajendran *et al.* (2011 ▶), and THAU (0.4 *versus* 0.15 MGy) by Kmetko *et al.* (2011 ▶). Here, we use averaged reported values for comparison. From re-analysing the cryotemperature data reported by Leal *et al.* (2011 ▶), the observed normalized half-dose values were between 12 and 23 MGy for seven different crystal systems.

### 4.4. Correlation with solvent content   

Joint analysis of the average normalized 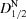 showed a remarkable increase in sensitivity with the crystal solvent content. To our knowledge, we present here only the second reliably documented systematic correlation of radiation sensitivity with a crystal property, the first being the dependence on absorbance described by Owen *et al.* (2006 ▶). The most obvious explanation of our data is that this dependence is due to the higher number of water-radiolysis radicals created per protein atom: 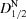 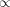 (1/solvent content) − 1. The relationship holds (approximately) for different crystal forms of insulin and trypsin. Considerations of concentration alone would suggest smaller differences in sensitivities between the monoclinic and trigonal lysozyme. Apparently, the lower lattice energies of high-solvent crystals also play a role. Such an explanation would also be consistent with the large variation in sensitivities between unrelated structures with similar solvent content.

### 4.5. Dose rate dependence   

Finally, our observation of the independence of the decay rates on the dose rate within the range between 0.5 and 300 kGy s^−1^ agrees well with the results by Warkentin *et al.* (2012 ▶) and Owen *et al.* (2012 ▶) made at similar temperature, dose rates and time scales of the experiments. Recently, dark progression of radiation damage on the second scale at 240 K (Warkentin *et al.*, 2012 ▶) and on the millisecond time scale at RT, as reported by Owen *et al.* (2012 ▶), was observed. In such ‘outrunning’ conditions, reliable and reproducible empirical characterization of radiation sensitivity will require considerably more sophisticated experimental procedures and data analysis protocols compared with the dose-dependent regime considered in this work.

## Supplementary Material

Supporting information file. DOI: 10.1107/S0909049512049114/xh5035sup1.pdf

## Figures and Tables

**Table 1:** Crystallographic parameters, experimental conditions and radiation damage parameter statistics Ref = Reference. No. = Number of crystals or crystal centerings. Res = Resolution. Ave = average; SD = standard deviation. Dose rate units: kGy s^−1^. β units: Å^2^ MGy^−1^. γ units: MGy^−1^. 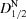 units: MGy.

				Unit cell	Space	Solvent content		Res	Dose rate		β		γ		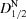	
Acronym	Protein	Source	Ref†	(Å, °)	group	(%)	No.	(Å)	Min	Max	Ave	SD	Ave	SD	Ave	SD
LYZM	Lysozyme	Hen egg-white	^*a*^	*a* = 28	*P*2_1_	34.0	7	1.9	0.4	225	15	0.5	1.0	0.2	0.56	0.07
				*b* = 63												
				*c* = 60												
				β = 90.2												
LYZT	Lysozyme	Hen egg-white	^*b*^	*a* = 79	*P*4_3_2_1_2	41.4	8	1.9	15	294	19	4	2.0	0.3	0.32	0.03
				*b* = 79												
				*c* = 38												
INSR	Insulin	Bovine pancreas	^*c*^	*a* = 83	*H*3	38.3	4	2.0	10	13	24	3	1.0	0.3	0.47	0.06
				*b* = 83												
				*c* = 109												
INSC	Insulin	Bovine pancreas	^*d*^	*a* = 79	*I*2_1_3	67.0	11	2.0	0.4	87	32	3	3.6	0.3	0.18	0.01
				*b* = 79												
				*c* = 79												
BPTTR	Trypsin	Bovine pancreas	^*e*^	*a* = 559	*P*3_1_21	36.5	8	2.0	8	40	13	1	1.1	0.3	0.58	0.10
				*b* = 559												
				*c* = 109												
BPTOH	Trypsin	Bovine pancreas	^*f*^	*a* = 55	*P*2_1_2_1_2_1_	45.0	3	1.7	9.6	44	14	3	1.7	0.2	0.40	0.04
				*b* = 59												
				*c* = 68												
BPTOL	Trypsin	Bovine pancreas	^*g*^	*a* = 63	*P*2_1_2_1_2_1_	56.9	10	2.2	11	14.3	20	4	2.8	0.4	0.24	0.03
				*b* = 64												
				*c* = 69												
bR	Bacterio-rhodopsin	*Halobacterium salinarium*	^*h*^	*a* = 62	*P*6_3_	51.1	3	3.0	1.1	124	29	6	1.0	0.06	0.42	0.05
				*b* = 62												
				*c* = 110												
TIM	Triosephosphate isomerase	*Leishmania mexicana*	^*i*^	*a* = 99	*C*2	52.1	1	2.5	111		33	–	1.6	–	0.3	–
				*b* = 53												
				*c* = 61												
				β = 118												
THER	Thermolysin	*Bacillus thermoproteo-lyticus*	^*j*^	*a* = 95	*P*6_1_22	53.8	4	2.8	17.4	20.4	28	5	0.9	0.2	0.44	0.02
				*b* = 95												
				*c* = 143												
THAU	Thaumatin	*Thaumatoccus daniellii*	^*k*^	*a* = 59	*P*4_1_2_1_1	56.8	4	2.5	11	16	20	2	1.3	0.2	0.43	0.03
				*b* = 59												
				*c* = 152												
FAE	SeMet-FAE‡	*Clostridium thermocellum*	^*l*^	*a* = 66	*P*2_1_2_1_2_1_	63.3	5	2.5	54	79	34	5	1.2	0.2	0.36	0.06
				*b* = 110												
				*c* = 114												
6hlno	6-hydroxy-L-nicotine oxidase	*Arthrobacter nicotinovorans*	^*m*^	*a* = 167	*P*432	69.7	3	3.5	26.5	28	215	30	3.2	0.3	0.07	0.01
				*b* = 167												
				*c* = 167												
LACV	L-protein polymerase *N*-terminal domain	La Crosse orthobunyavirus	^*n*^	*a* = 125	*P*6_1_22	69.9	5	3.9	27	113	142	20	5.7	1.0	0.08	0.003
				*b* = 125												
				*c* = 167												
TvNiR	Cytochrome *c* nitrite reductase	*Thioalkalivibrio nitrati­reducens*	^*o*^	*a* = 197	*P*2_1_3	77.7	3	2.6	30		11	1	0.6	0.1	0.91	0.09
				*b* = 197												
				*c* = 197												

† ^*a*^Hogle *et al.* (1981 ▶). ^*b*^Blake *et al.* (1965 ▶). ^*c*^Smith *et al.* (2005 ▶). ^*d*^Nanao *et al.* (2005 ▶). ^*e*^Bode & Huber (1978 ▶). ^*f*^Marquart *et al.* (1983 ▶). ^*g*^Bartunik *et al.* (1989 ▶). ^*h*^Borshchevskiy *et al.* (2011 ▶). ^*i*^Alahuhta & Wierenga (2010 ▶). ^*j*^Mueller-Dieckmann *et al.* (2007 ▶). ^*k*^Charron *et al.* (2002 ▶). ^*l*^Prates *et al.* (2001 ▶). ^*m*^Kachalova *et al.* (2010 ▶). ^*n*^Reguera *et al.* (2010 ▶). ^*o*^Boyko *et al.* (2006 ▶).

‡ Selenomethionine labeled feruloyl esterase module of xylanase 10B.

## Footnotes

1 Supplementary data for this paper are available from the IUCr electronic archives (Reference: XH5035). Services for accessing these data are described at the back of the journal.
