# Supplementary material for: A survey of global radiation damage to 15 different protein crystal types at room temperature: a new decay model
Source: J Synchrotron Radiat. 2012 Dec 6;20(Pt 1):14–22. doi: 10.1107/S0909049512049114 (PMC3943537; doi:10.1107/S0909049512049114)
Supplement: Supplementary file 1 [file s-20-00014-sup1.pdf]

Supplementary Figure

Variation in the scaling parameters and total scattering intensity with dose for fifteen different protein crystal types at room temperature. All sets of measurements are shown; each of the sets of symbols is a different series of wedge measurements. (a) B-factors: the solid line represents an approximation according to eq. 2 using the best fit of Bo and β. (b) *scale* factors divided by the *constant* (eq. 3), the solid line represents $exp(-\gamma^2 D^2)$ using the best fit value of γ. (c) Total scattered intensity, estimated by intensity summation (dots) and calculated according to Eq. (4) (solid lines), using the best fit Bo, β and γ as in (a) and (b).

Lysozyme monoclinic

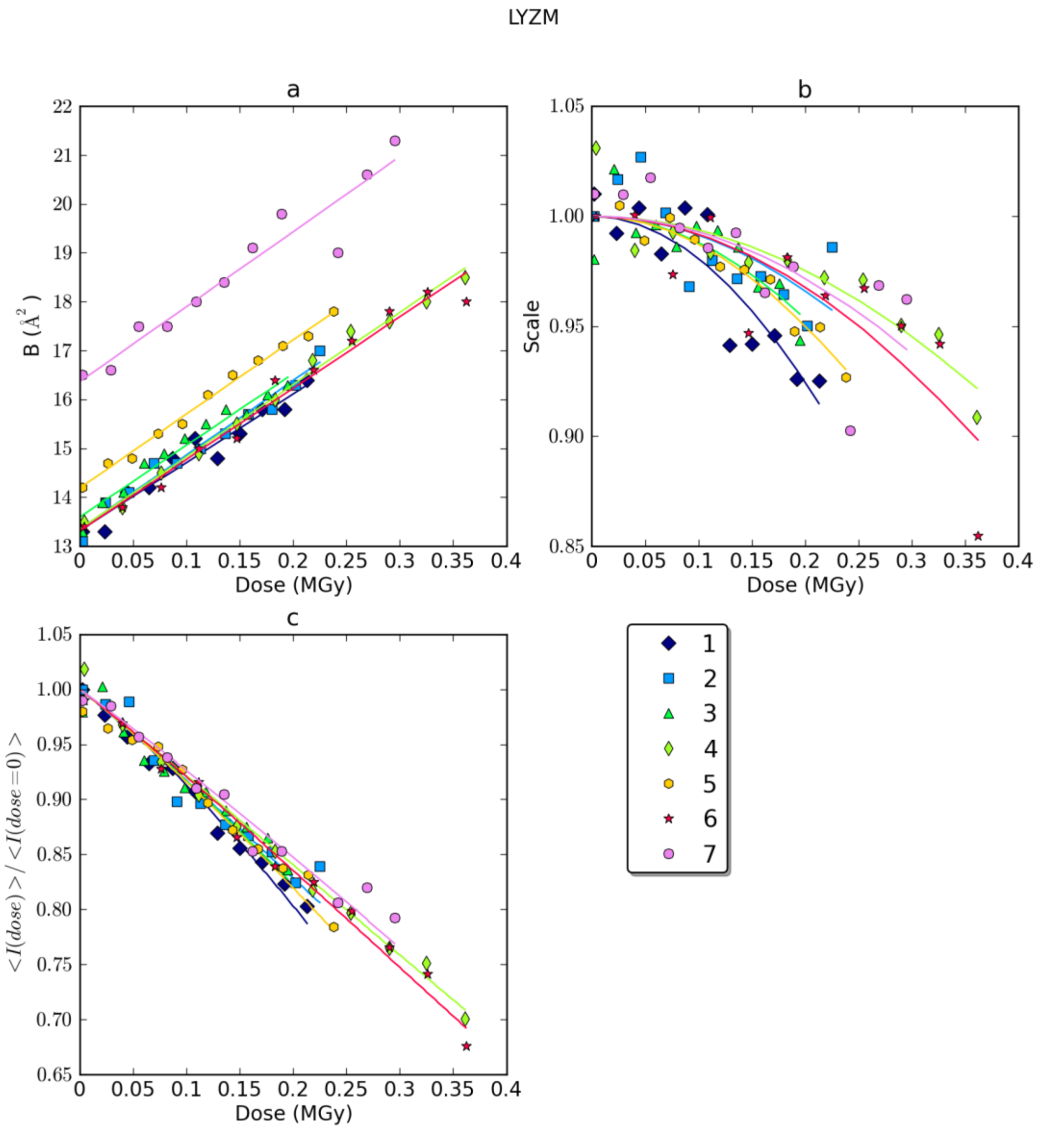

Lysozyme tetragonal

LYZT

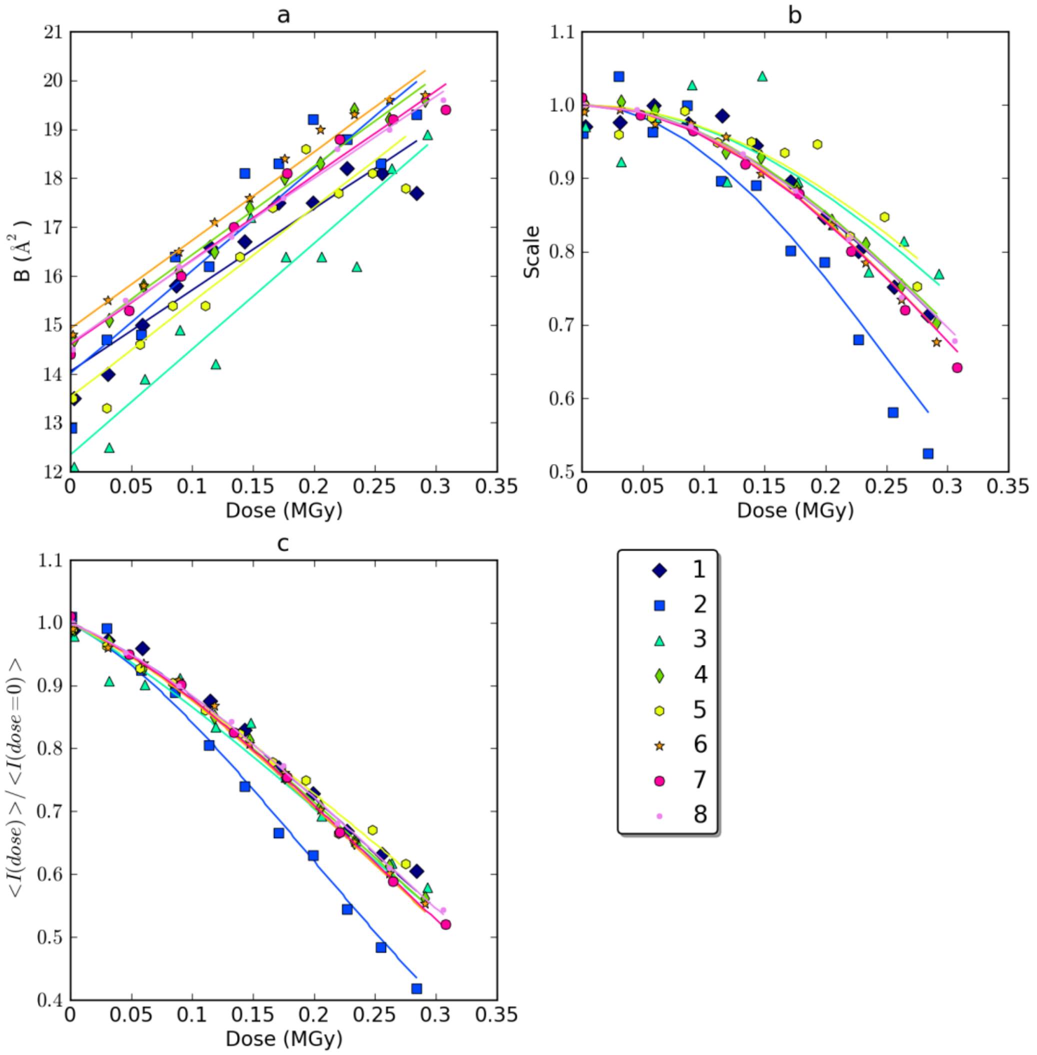

Insulin rombohedral

INSR

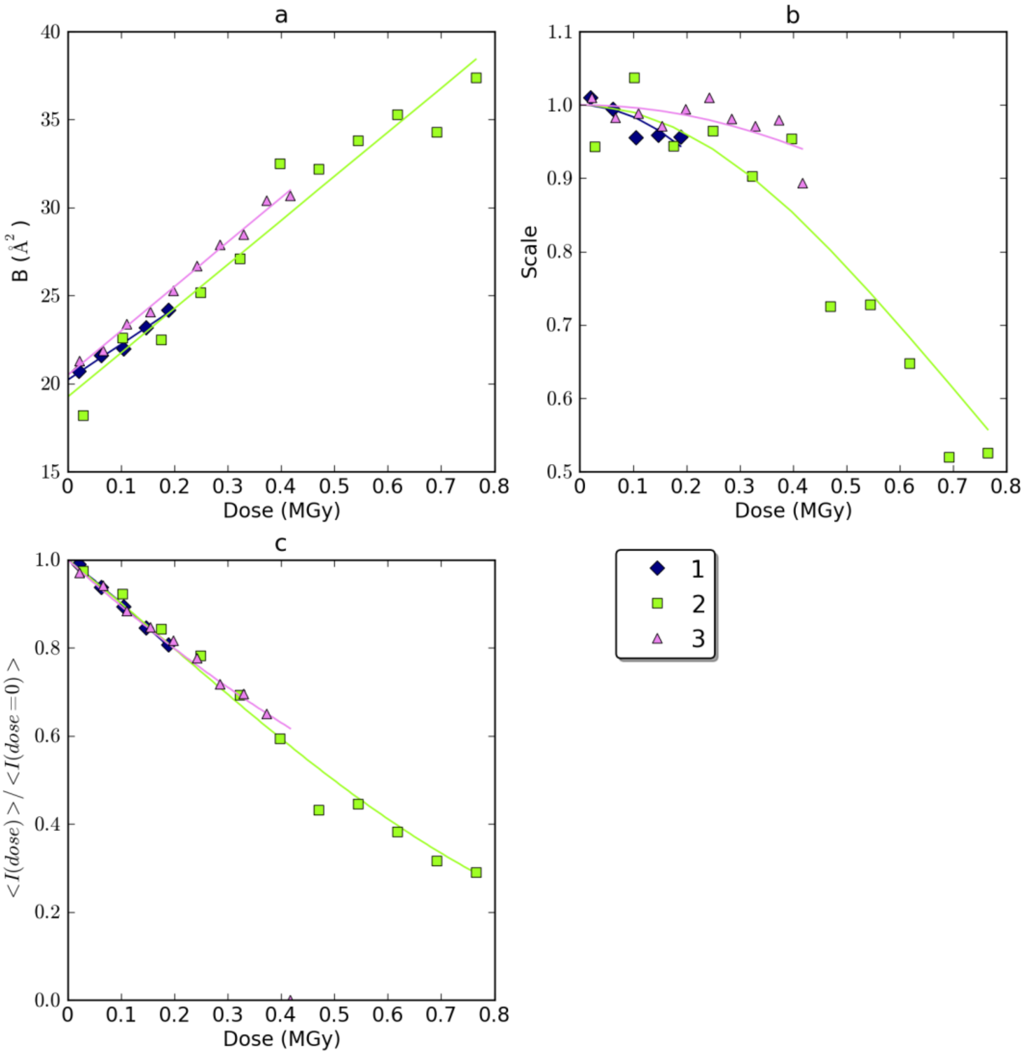

Insulin cubic

INSC

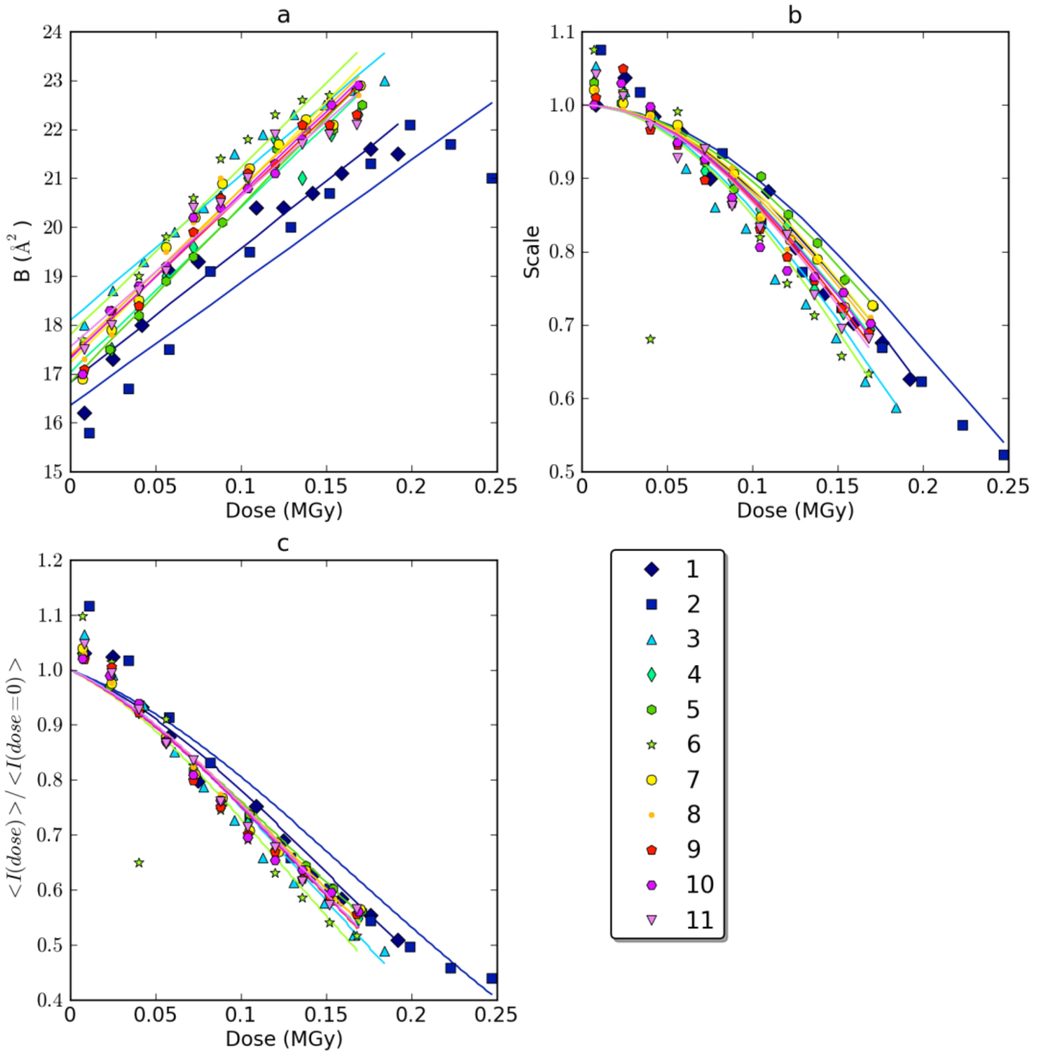

Bovine pancreatic trypsin, trigonal

BPTTR

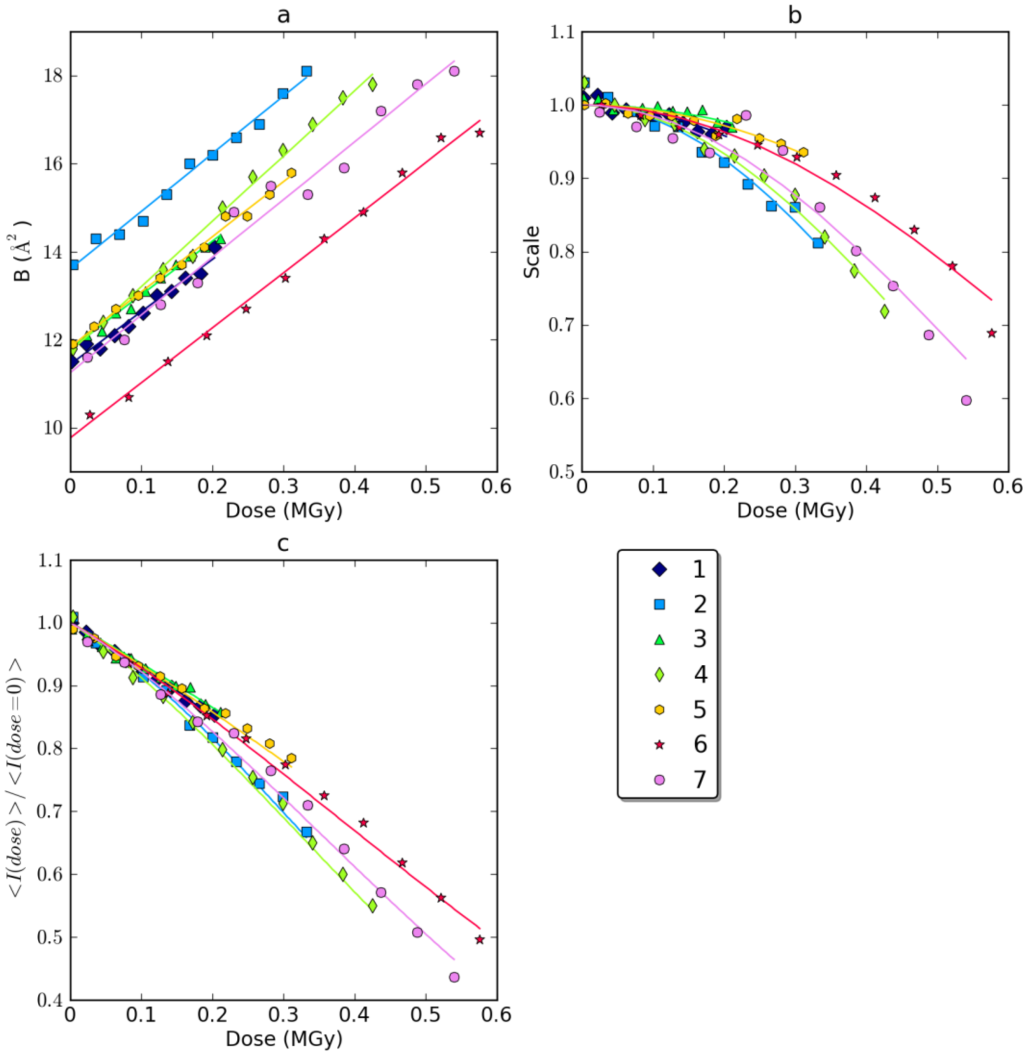

Bovine pancreatic trypsin, orthorhombic, high density form

BPTOH

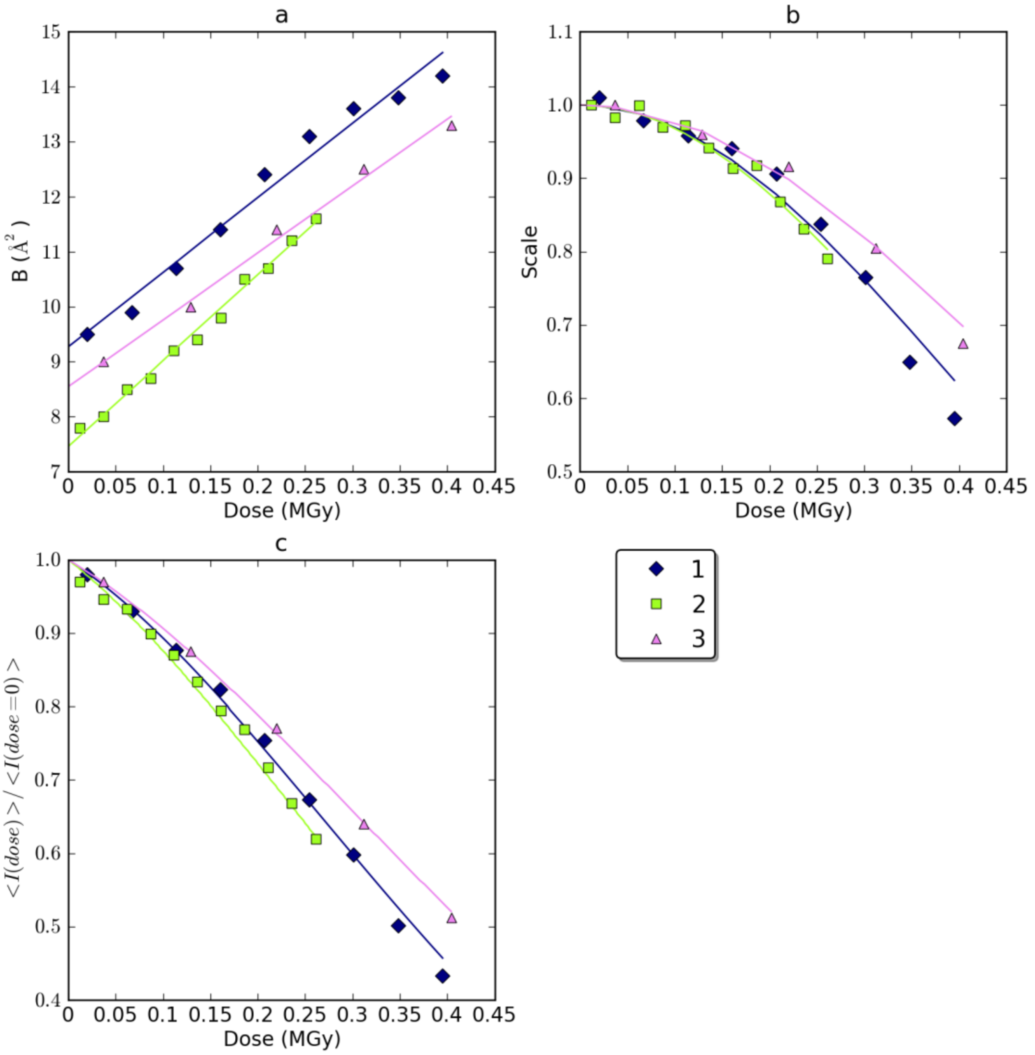

Bovine pancreatic trypsin, orthorhombic, low density form

BPTOL

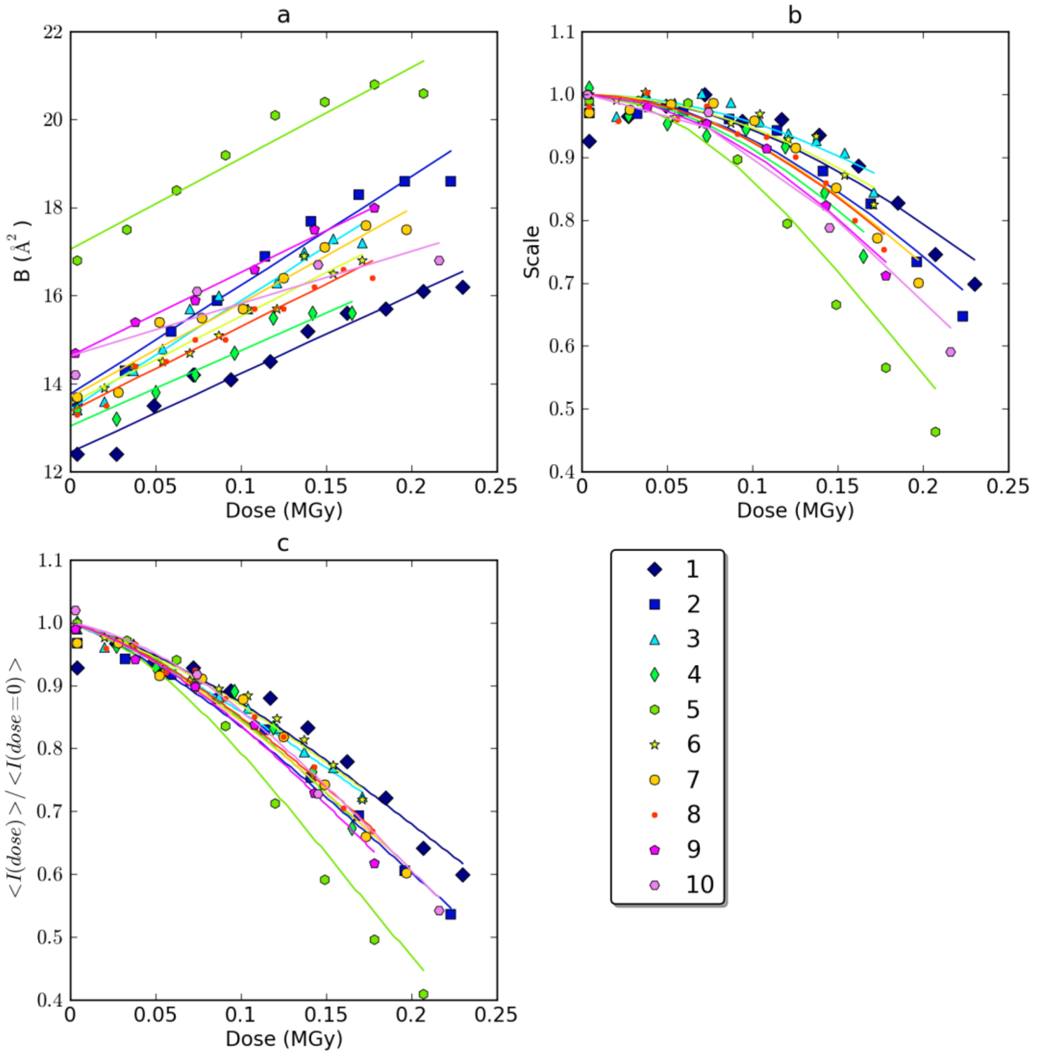

Bacteriorhodopsin

bR

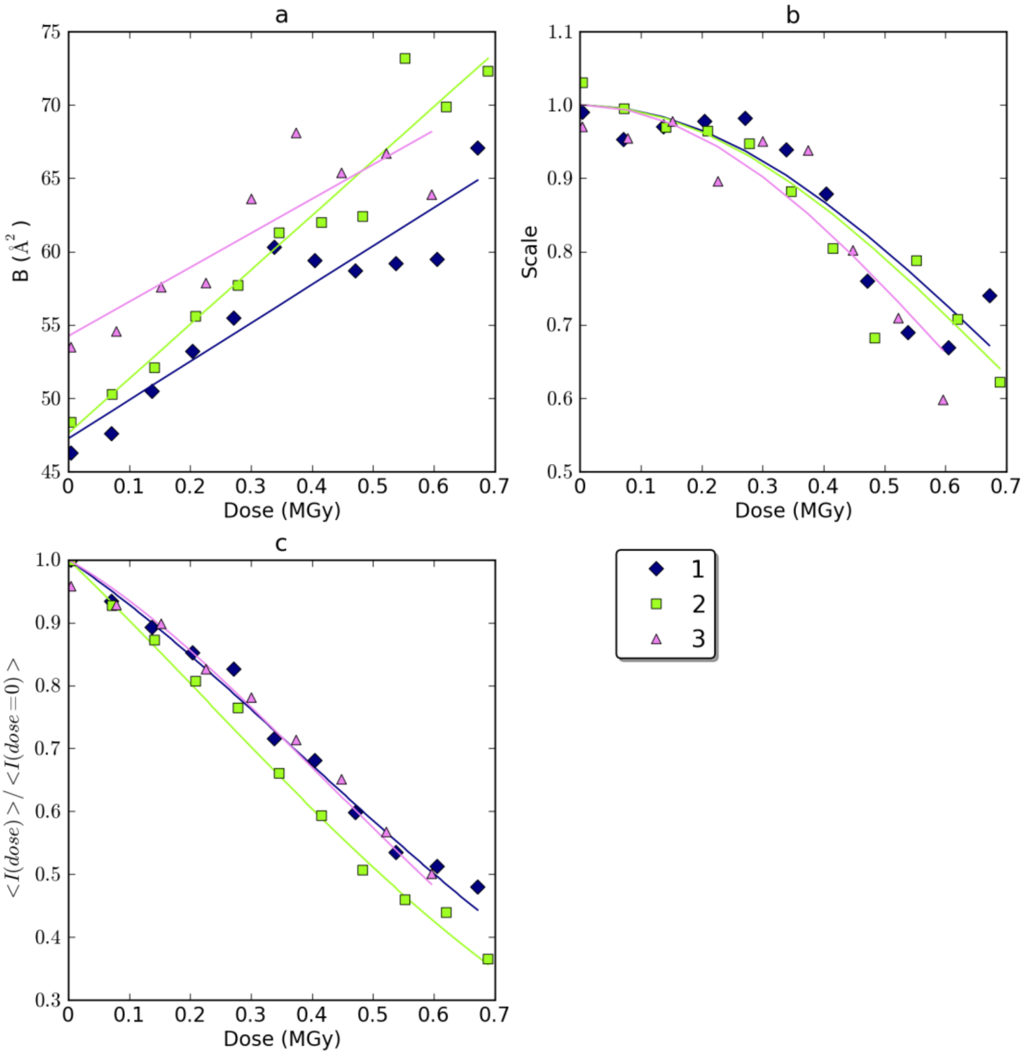

Triosephosephosphate isomerase

TIM

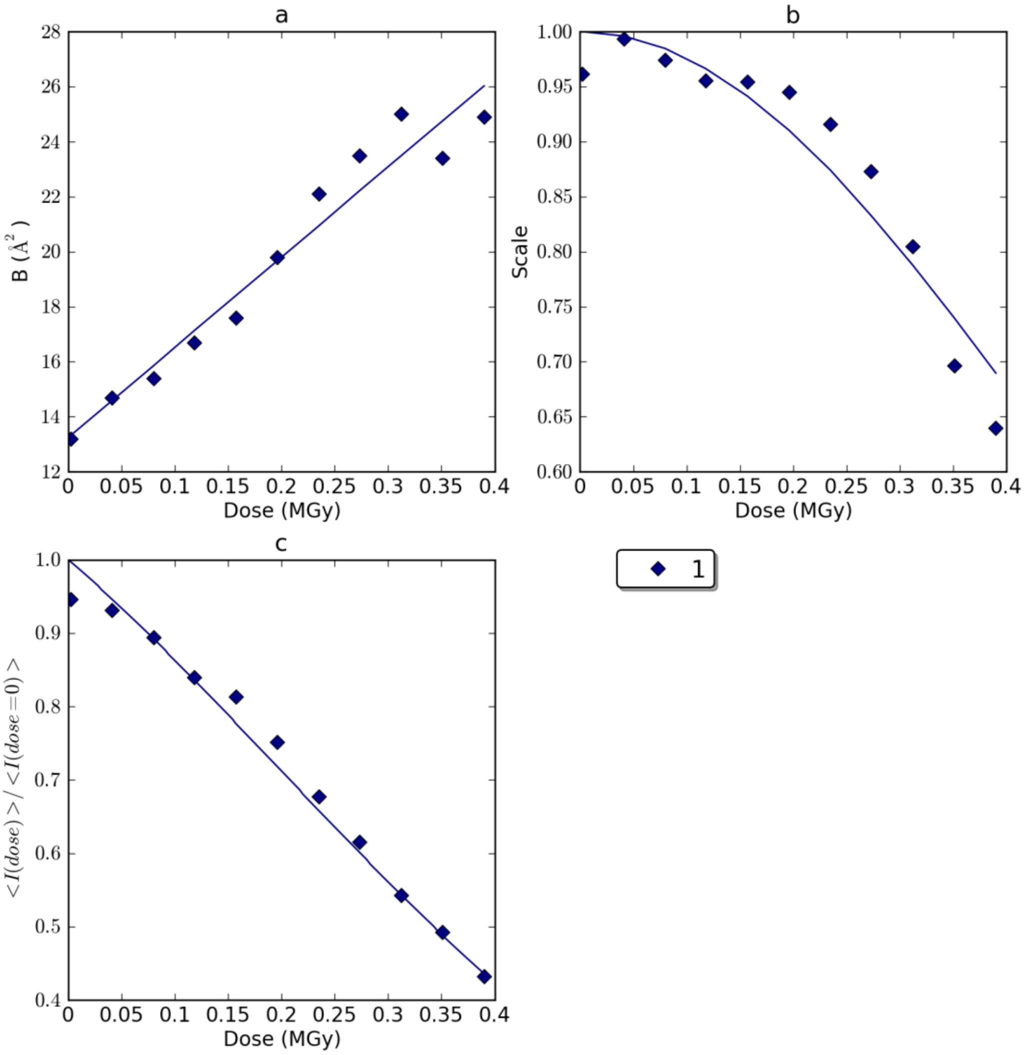

Thermolysin

THER

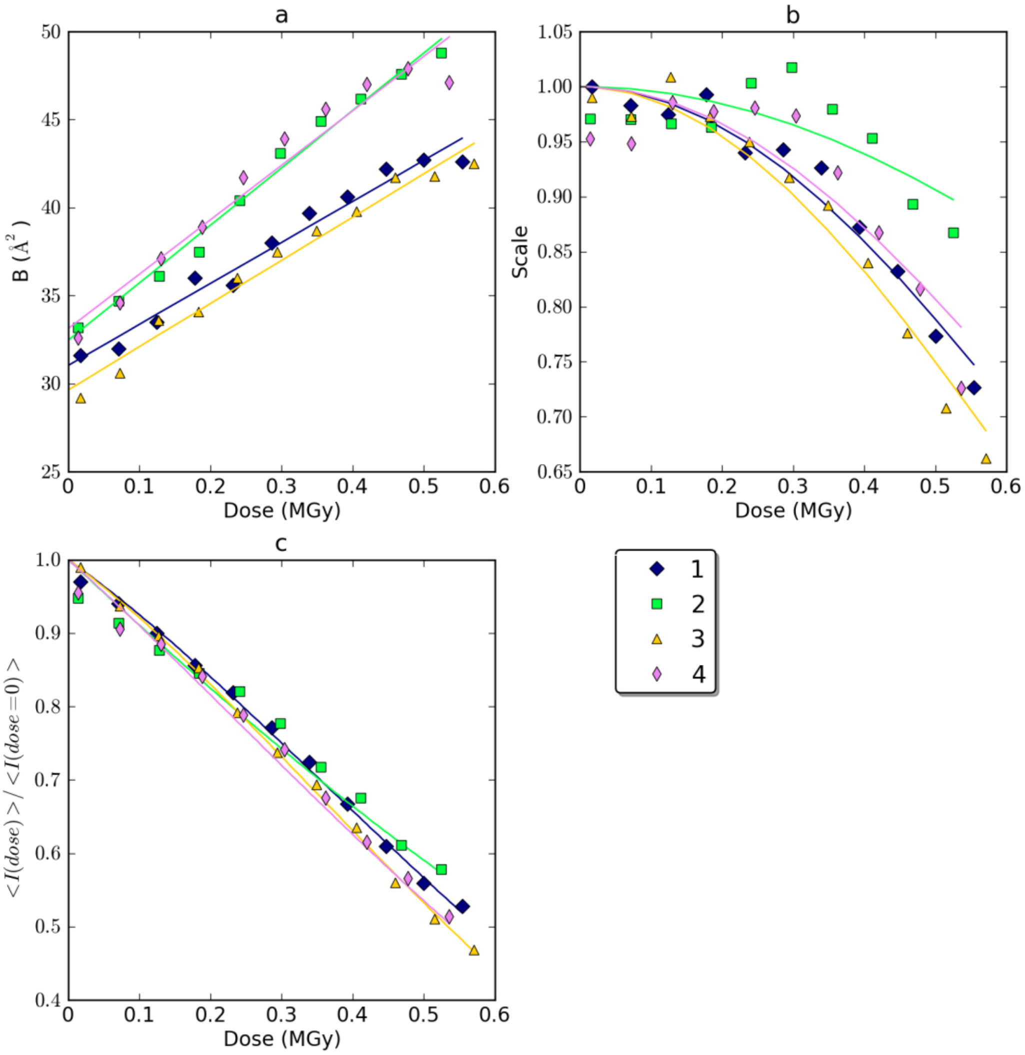

Thaumatin

THAU

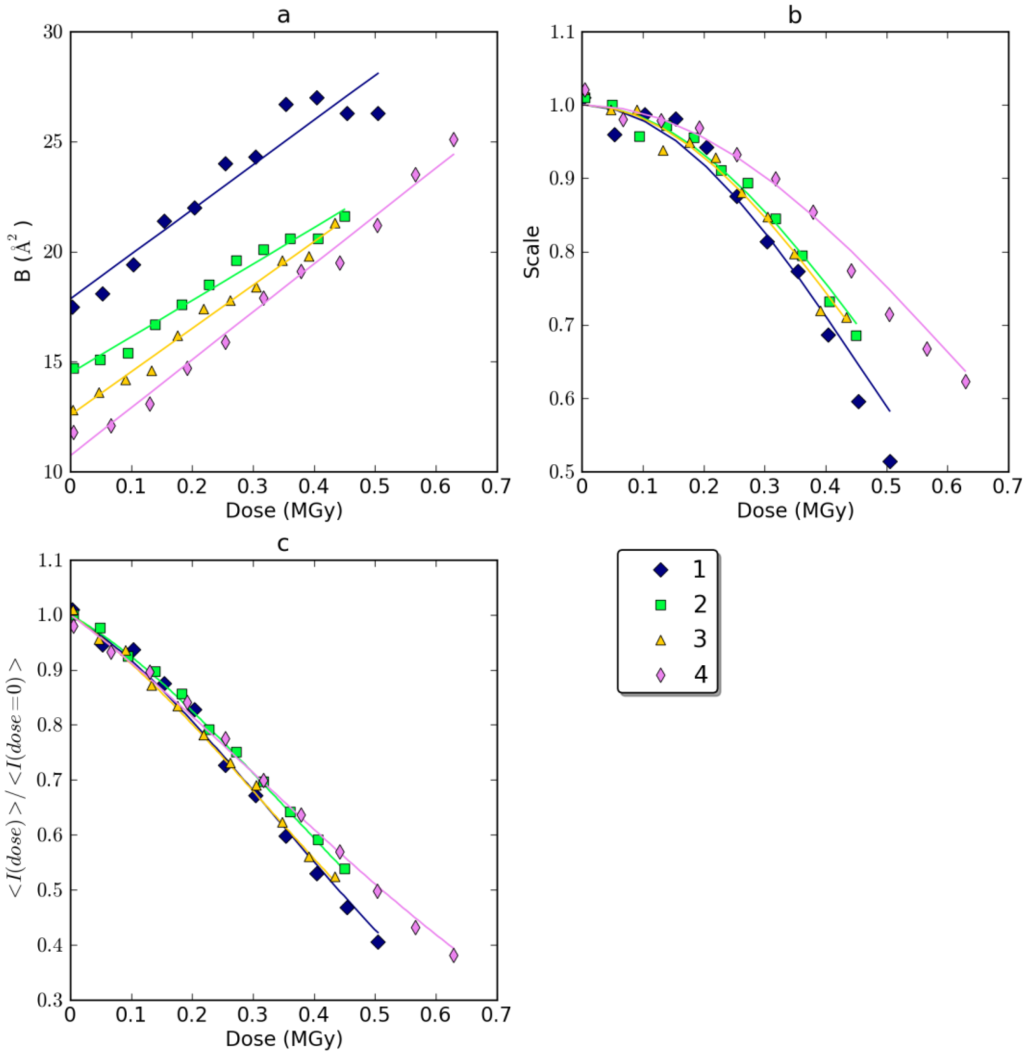

Feruloyl esterase module of xylanase

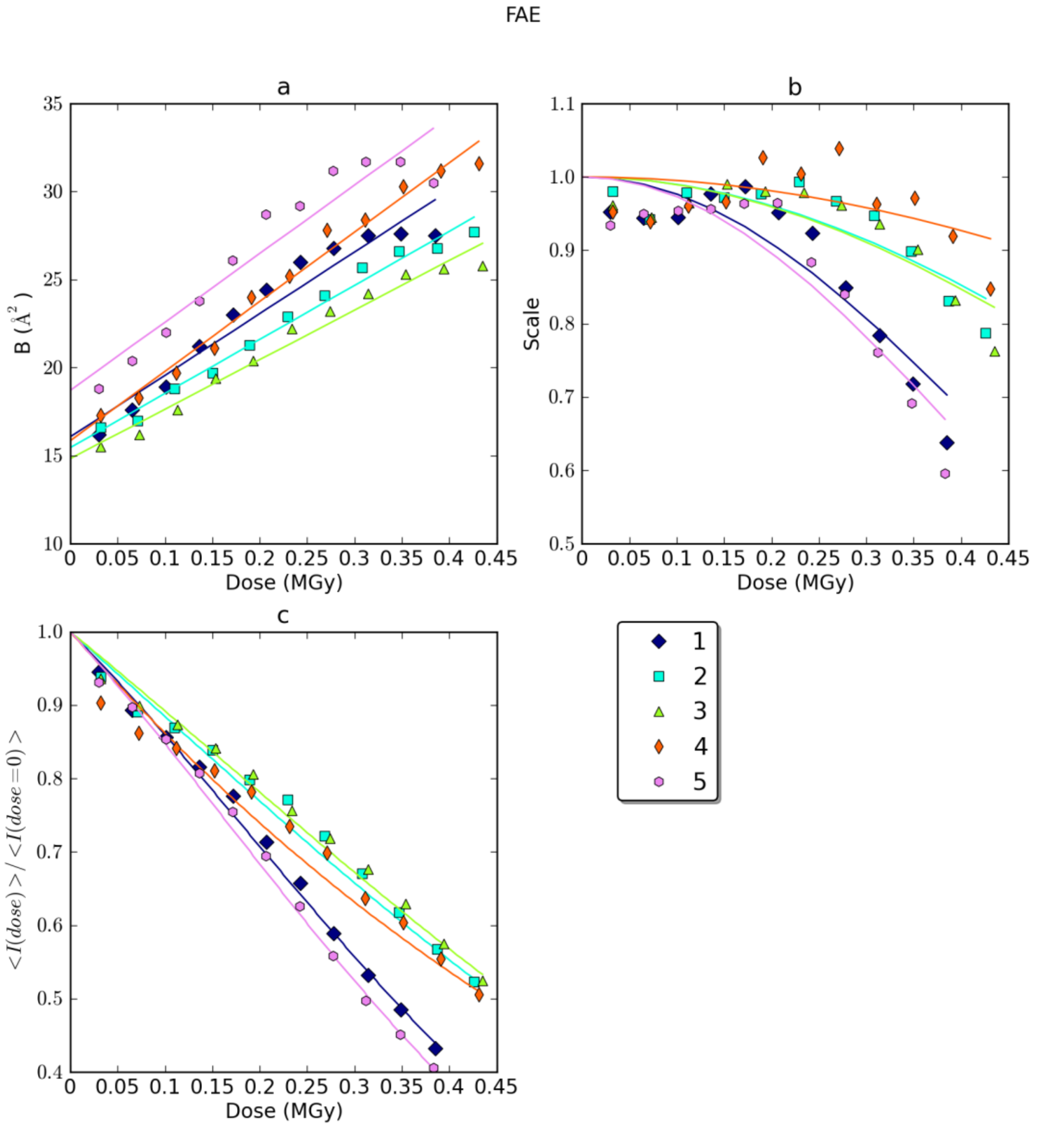

6-hydroxy-L-nicotine oxidase

6HLNO

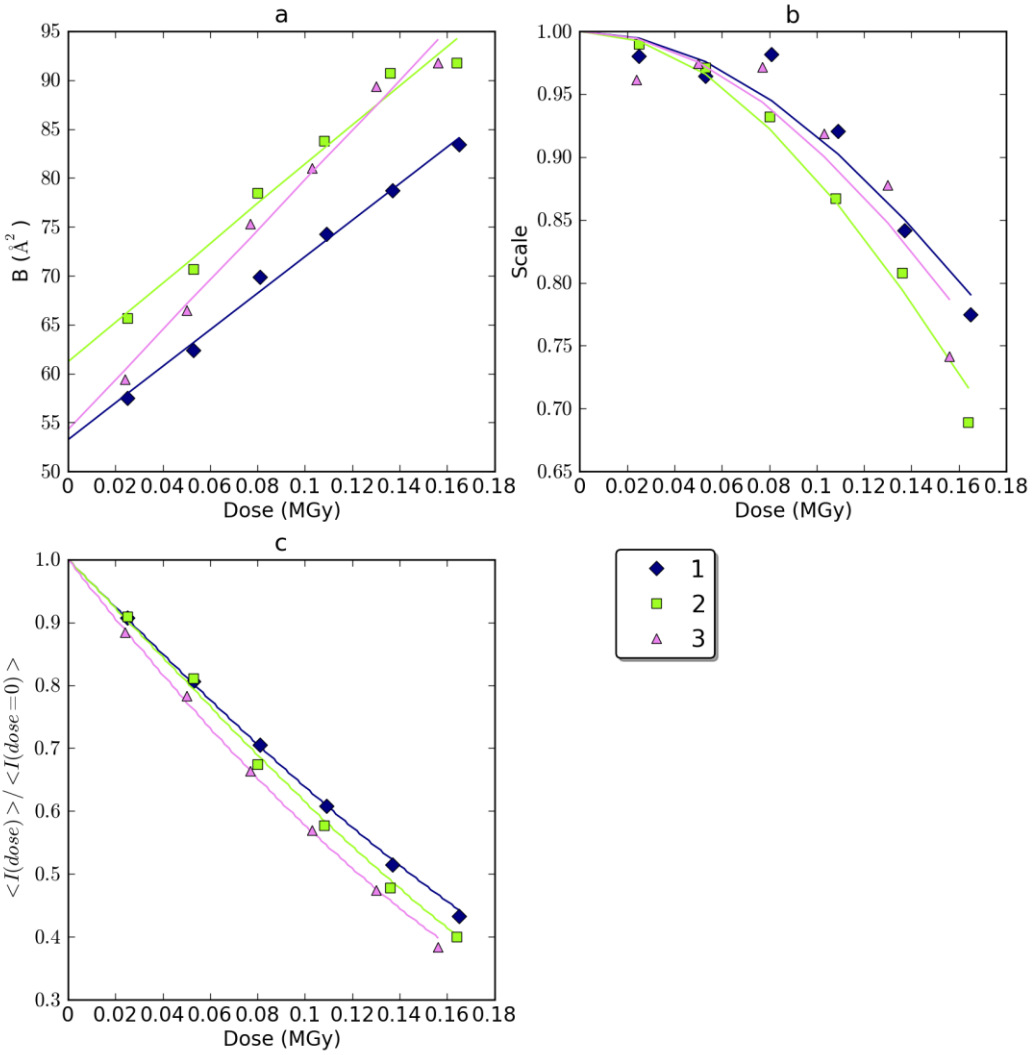

La Crosse orthobunyavirus L-protein polymerase, N-terminal domain

LACV

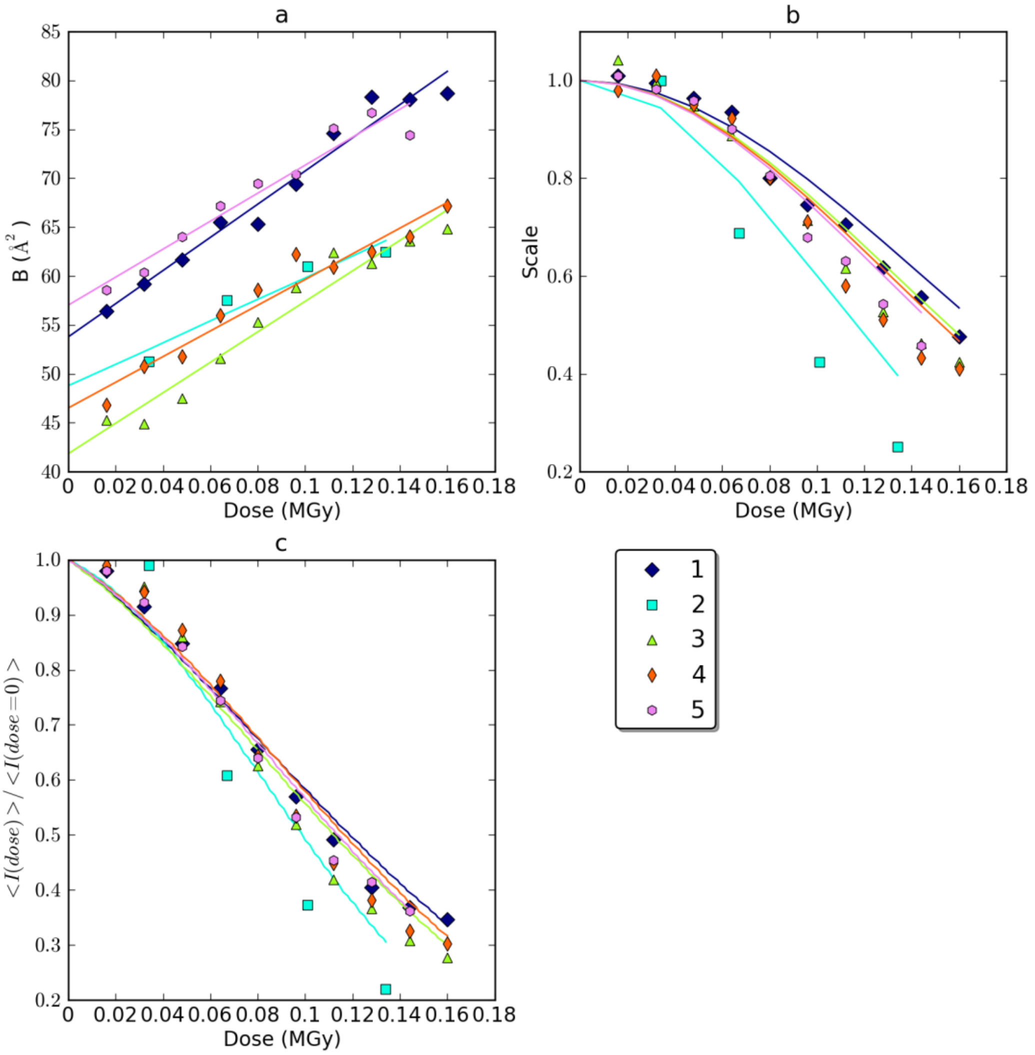

Thioalkalivibrio nitratireducens Cytochrome c nitrite reductase

TvNiR

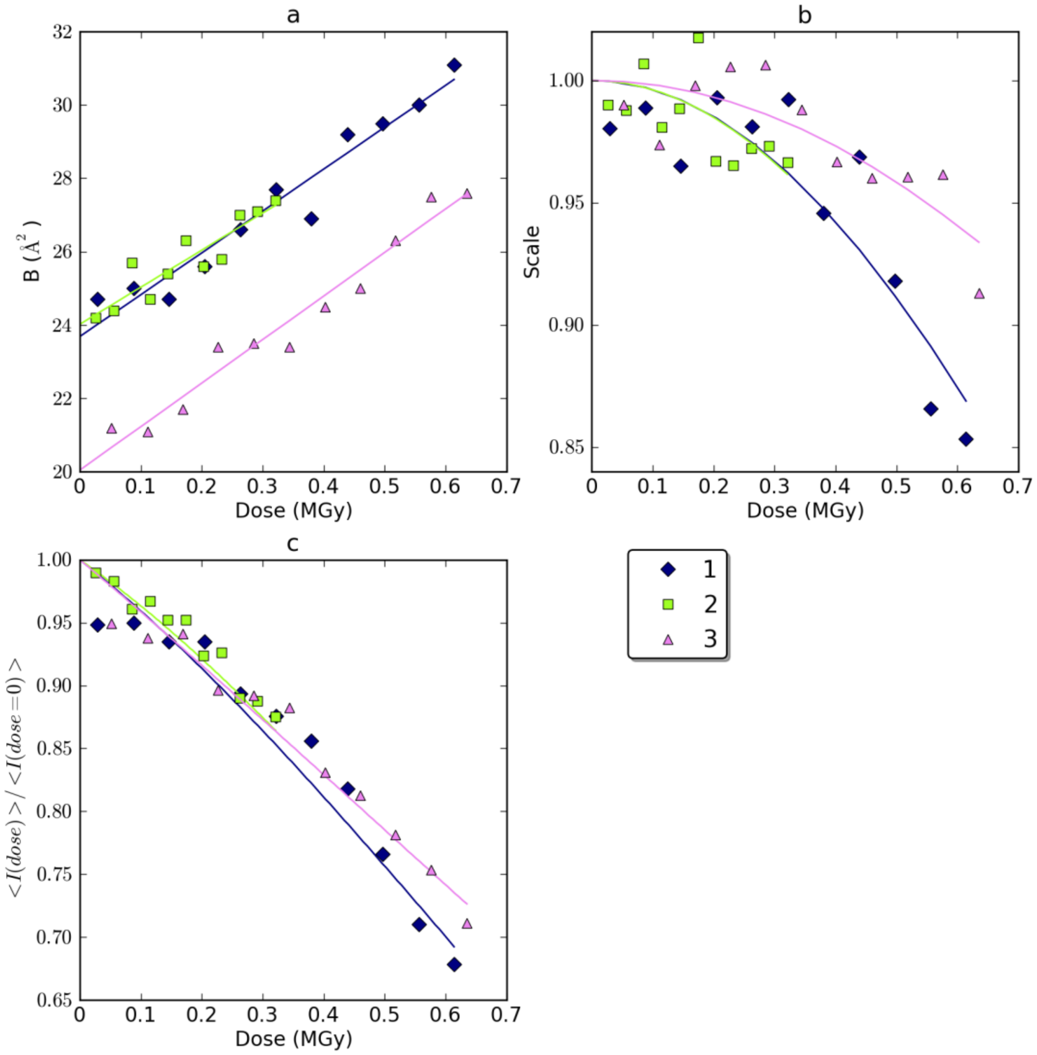

Supplementary Table
Data set resolution, initial B-factor (Bo), radiation damage model parameters β and γ, half-dose $D_{1/2}$ and normalized half-dose $D^N_{1/2}$ for all data sets.

.

Protein/Crystal number	Resolution **Å**	B-factor **Å^2**	**β** **Å^2 MGy^{-1}**	γ **MGy^{-1}**	$D^H_{1/2}$ **MGy**	**$D_{1/2}$** **MGy**
Lysozyme monoclinic (LYZM)						
1	1.90	13.3	13.95	1.4	0.452	0.446
2	1.90	13.3	15.23	0.93	0.582	0.568
3	1.90	13.6	14.68	1.1	0.53	0.521
4	1.95	13.4	14.76	0.79	0.65	0.633
5	1.90	14.2	15.12	1.13	0.514	0.508
6	1.90	13.3	14.57	0.9	0.602	0.588
7	2.05	16.4	15.32	0.85	0.615	0.629
Lysozyme tetragonal (LYZT)						
1	1.90	14.1	16.57	2.01	0.328	0.326
2	1.90	14.0	21.02	2.6	0.255	0.254
3	1.90	12.4	21.57	1.81	0.333	0.325
4	1.90	14.6	18.21	1.99	0.324	0.323
5	1.90	13.5	19.38	1.76	0.35	0.345
6	1.90	14.9	18.01	2.07	0.315	0.314
7	1.90	14.6	17.3	2.08	0.317	0.316
8	1.90	14.6	16.82	2.01	0.328	0.327
Insulin rombohedral (INSR)						
1	2.00	20.2	20.25	1.27	0.43	0.447
2	2.35	19.3	25.04	1	0.448	0.498
3	2.00	20.5	25.16	0.6	0.545	0.584
Insulin cubic (INSC)						
1	2.00	16.8	27.43	3.49	0.187	0.198
2	2.00	16.4	25.06	3.18	0.204	0.216
3	2.00	18.1	29.68	3.94	0.171	0.179
4	2.00	17.0	33.71	3.63	0.176	0.186
5	2.00	16.8	35.99	3.32	0.187	0.198
6	2.00	17.8	34.23	4.04	0.16	0.168
7	2.00	17.2	35.52	3.47	0.181	0.191
8	2.00	17.4	33.72	3.55	0.181	0.191
9	2.00	17.3	32.81	3.71	0.176	0.185
10	2.00	17.3	33.65	3.66	0.177	0.187
11	2.00	17.6	30.64	3.76	0.172	0.181
Bovine pancreatic trypsin, trigonal (BPTTR)						
1	2.00	11.4	11.93	1.04	0.587	0.579

2	2.00	13.6	13.06	1.38	0.463	0.463
3	2.00	11.8	12.23	0.75	0.721	0.707
4	2.00	11.7	14.82	1.3	0.468	0.462
5	2.00	11.8	12.52	0.84	0.665	0.654
6	2.00	11.3	13.11	1.21	0.512	0.505
7	2.00	9.8	12.5	0.96	0.609	0.592
Bovine pancreatic trypsin, orthorhombic, high density form (BPTOH)						
1	1.70	9.3	13.54	1.74	0.385	0.365
2	1.70	7.5	15.61	1.8	0.364	0.339
3	1.70	8.6	12.15	1.48	0.446	0.42
Bovine pancreatic trypsin, orthorhombic, low density form (BPTOL)						
1	2.20	12.4	17.85	2.4	0.281	0.286
2	2.20	13.8	24.7	2.73	0.237	0.243
3	2.20	13.4	24.71	2.14	0.284	0.292
4	2.20	13.0	17.18	3.01	0.232	0.232
5	2.20	17.1	20.63	3.83	0.183	0.183
6	2.20	13.6	19.8	2.34	0.28	0.287
7	2.20	13.7	21.43	2.8	0.24	0.245
8	2.20	13.4	19.22	2.82	0.243	0.248
9	2.20	14.6	19.02	3.13	0.224	0.224
10	2.20	14.6	11.91	3.16	0.222	0.222
Bacteriorhodopsin (bR)						
1	3.00	47.3	26.21	0.94	0.45	0.6
2	3.00	47.6	37.08	0.97	0.359	0.512
3	3.00	54.3	23.35	1.07	0.447	0.579
Triosephosephosphate isomerase (TIM)						
1	2.50	13.2	32.8	1.56	0.312	0.342
thermolysin (THER)						
1	2.80	31.0	23.28	0.97	0.47	0.576
2	3.00	32.5	32.62	0.63	0.444	0.634
3	2.90	29.7	24.53	1.07	0.437	0.534
4	2.95	33.1	30.86	0.93	0.411	0.54
Thaumatin (THAU)						
1	2.80	17.9	20.31	1.46	0.394	0.44
2	2.50	14.5	16.54	1.32	0.449	0.48
3	2.50	12.6	19.69	1.36	0.412	0.442
4	2.60	10.7	21.76	1.07	0.463	0.512
Feruloyl esterase module of xylanase (FAE)						
1	2.50	16.1	34.98	1.54	0.305	0.34
2	2.50	15.5	30.72	1	0.4	0.453
3	2.50	14.8	28.13	1.03	0.417	0.469
4	2.50	15.9	39.5	0.69	0.374	0.443
5	2.50	18.7	38.84	1.65	0.28	0.316
6-hydroxy-L-nicotine oxidase (6HLNO)						

1	3.60	53.3	186.9	2.94	0.081	0.144
2	3.70	61.2	201.59	3.53	0.073	0.133
3	3.80	54.3	255.2	3.14	0.061	0.123
La Crosse orthobunyavirus L-protein polymerase, N-terminal domain (LACV)						
1	3.90	53.8	170.06	4.96	0.075	0.119
2	3.95	48.8	110.6	7.17	0.077	0.098
3	3.80	41.9	156.18	5.37	0.077	0.112
4	3.80	46.5	131.68	5.47	0.083	0.117
5	3.80	57.0	143.46	5.58	0.079	0.114
Thioalkalivibrio nitratireducens Cytochrome c nitrite reductase (TvNiR)						
1	2.60	23.7	11.4	0.61	0.841	0.961
2	2.60	24.0	10.11	0.62	0.877	0.99
3	2.60	20.0	11.87	0.41	1.006	1.182
